# Prevalence and patterns of multimorbidity among tuberculosis patients in Brazil: a cross-sectional study

**DOI:** 10.1186/1475-9276-12-61

**Published:** 2013-08-20

**Authors:** Bárbara Reis-Santos, Teresa Gomes, Laylla R Macedo, Bernardo L Horta, Lee W Riley, Ethel L Maciel

**Affiliations:** 1 Lab-Epi UFES Laboratory of Epidemioly of Universidade Federal do Espírito Santo, Av. Marechal Campos 1468 – Maruípe, Vitória, ES, Brazil; 2 Post-Graduate Programme in Epidemiology, Universidade Federal de Pelotas, Pelotas, Brazil; 3 Division of Infectious Disease and Vaccinology, School of Public Health, University of California, Berkeley, CA, USA

**Keywords:** Tuberculosis, Multimorbidities, Inequity, Social determinants, Hierarchical models

## Abstract

**Introduction:**

The number of subjects with tuberculosis (TB) presenting with co-occurrence of multiple chronic medical conditions, or multimorbidity (MM) is increasing in Brazil. This manuscript aimed to characterize subjects with TB, according to their MM status and to analyse factors associated with TB treatment outcomes.

**Methods:**

This is a cross-sectional study that included 39,881 TB subjects reported in Brazil, in 2011. MM were defined as any (two or more) occurrence of chronic medical conditions in a TB patient (TB–MM). Data analysis was performed by hierarchical logistic regression models comparing TBMM with those with only TB.

**Results:**

Of the reported TB cases in 2011, 454 (1.14%) had MM. The subjects in the age group 40–59 years (OR: 17.89; 95% CI, 5.71-56.03) and those ≥ 60 years (OR: 44.11; 95% CI, 14.09-138.06) were more likely to develop TB–MM. The TB–MM subjects were less likely to be male (OR: 0.63; 95% CI, 0.52-0.76), institutionalized (OR: 0.59; 95% CI, 0.23-0.80) and live in rural areas (OR: 0.63; 95% CI, 0.42-0.95). Death from causes other than TB was higher among TB–MM subjects (OR: 1.76; 95% CI, 1.36-2.28). Of 454 TB–MM subjects 302 (66.5%) were cured and 152 (33.5%) were not cured. The odds of not being cured was 1.55 (95% CI, 1.04-2.32) among males, 2.85 (95% CI, 1.12-7.28) among institutionalized subjects, and 3.93 (IC 95%, 1.86-8.30) among those who were infected with HIV. TB retreatment after previous abandonment (OR: 7.53; 95% CI, 2.58-21.97) and transfer from a treatment site (OR: 2.76; 95% CI, 1.20-6.38) were higher for subjects not cured compared to those who were cured.

**Conclusions:**

While TB is well recognized to be a disease engendered by social inequity, we found that even among TB patients, those who have MM have greater inequity in terms of socioeconomic status and adverse clinical outcomes. Addressing the problem of TB and TB–MM requires a multisectorial approach that includes health and social service organizations.

## Introduction

Worldwide, the proportion of subjects with multiple coexistent medical conditions, or multimorbidity (MM), is increasing [1]. MM is defined as co-occurrence of multiple diseases or medical conditions in the same individual [2].

Tuberculosis (TB) is an important chronic infectious disease problem, which has a strong social determination [3,4]. In 2010, it was the cause of death of 4,600 people in Brazil and fourth most common infectious disease cause of death [5]. Because TB is a chronic infectious disease, it frequently co-occurs with other chronic medical conditions [6,7].

The prevalence of MM has often been investigated in developed countries [8-11], but available literature on MM in developing countries is limited. The determinants of MM [age, sex, area deprivation (an area’s potential for health risk from ecological concentration of poverty, unemployment, economic disinvestment, and social disorganisation [12]) and difficulty in access to health services] have already been reported [13]. The lack of access to health services, especially to primary care services, where most health care is provided, may generate a wide range of problems at the individual level, such as lack of or delayed diagnosis, disease complications, and delay in treatment, which, then can affect the health of the entire population where such individuals reside [13]. Reliable estimates of MM in TB subjects can prepare the health services to better manage the health problem of their patients living in resource-limited conditions.

This study aimed to characterize subjects with TB, according to their MM status and to analyze factors associated with TB treatment outcomes.

## Patients and methods

In Brazil, TB cases are registered in the National Notification System (SINAN) from investigation and follow-up medical chart reviews of a TB case, and it is the principal instrument in the country for collecting and analysing national TB data [14,15].

This is a cross-sectional study based on SINAN database, including TB cases reported in Brazil in 2011. Those subjects with missing information on TB treatment outcome were excluded.

The subjects were classified as “TB and MM subjects” (TB – MM subjects) if they had both TB and MM, and “TB without MM subjects” (TB subjects) if they had no MM. MM was defined as any (two or more) occurrence of the following medical conditions in a TB patient: arthritis, cancer, diabetes mellitus, hypertension, heart disease, obstructive lung disease and psychiatric problems [2].

TB treatment outcomes were classified as “cured” and “not cured”. The “not cured” group included the following SINAN categories: abandonment, death from TB, death from other cause than TB and development of multidrug resistant TB (MDR TB).

The socio-demographic covariates evaluated were: age (< 20 years, 20 – 39 years, 40 – 59 years and ≥ 60 years), gender (female, male), skin color (white, non-white), schooling (< 4 years, 4 to 8 years, > 8 years), area of residence (urban, rural) and institutionalization status [no, yes (prison, shelter, orphanage, psychiatric hospital)].

The covariates related to TB features included type of treatment (new TB case, relapse, return after abandonment, transferred and unknown) and TB presentation (pulmonary, extra pulmonary, pulmonary + extra pulmonary), tuberculin skin test (negative, positive if higher than 10+ mm), existence of chest X-ray suspicious for TB, result of initial bacilloscopy test, result of initial culture examination and result of initial histopathologic examination.

Supervision status under directly observed therapy (DOT) and the occupational setting of TB transmission (TB acquired at workplace mainly determined by inadequate environments or conditions of work) were included as covariates also.

### Data analysis

Initially, we compared TB – MM subjects with TB subjects according to socio-demographic features, health-related history and TB clinical features. Then the treatment outcome of TB – MM subjects was evaluated according to socio-demographic features, health-related history and TB clinical features. Pearson chi-square test or likelihood-ratio chi-square test, when more than twenty percent of categories had less than five observations, were used to compare proportions. Covariates associated (p ≤ 0.10) with the outcome of interest were included in a hierarchical logistic regression model.

The covariates were grouped into a hierarchy of categories, ranging from distal determinants to proximate ones [16]. The level 1 included socioeconomic characteristics; level 2 variables evaluated the environmental characteristics; level 3 variables were related to health conditions associated; level 4 variables assessed TB clinical features; and level 5 variables were TB outcome/care.

As the purpose was to identify a parsimonious model to explain the data, in each set the confounders were selected through backward elimination, according to an alpha level of 0.10 (p ≤ 0.10). Thus, the covariates were evaluated after adjustment for confounders in the same set or in hierarchically superior sets. This approach allows researchers to quantify the contribution of each level of adjustment to understand the model building strategy as well as to interpret the independent associations [16].

All analyses were conducted with the Stata® statistical package, Version 9 (Stata Corp, College Station, TX, 2001).

The Institutional Review Board of the Federal University of Espirito Santo, Brazil, under number 121/06, approved this project.

## Results

In 2011, 454 (1.14%; 95% CI, 1.04 – 1.25%) patients among 39,881 treated cases of TB had MM as reported by SINAN.

The study was conducted in two stages. In the first stage, we analyzed 39,881 subjects comparing TB subjects with and without MM; in the second stage, we analyzed TB treatment outcome of TB subjects with MM.

Among the 454 subjects with TB-MM, 383 had diabetes mellitus; 283 had hypertension; 129 had psychiatric disease; 54 had cardiovascular disease; 32 had cancer; 32 had chronic pulmonary obstructive disease; and 9 were reported to have arthritis. Accordingly, 93% of subjects had two, 6% had three, and 1% had four comorbidities.

Gender distribution was different between the groups; the prevalence of TB – MM was 1.0% among males and 1.5% among females (p < 0.001), and the proportion of subjects with TB – MM increased with age (< 20 years: 0.1%; 20 – 39 years: 0.3%; 40 – 59 years: 1.5% and ≥ 60 years: 3.6%; p < 0.001). Low school level (up to eight years of study) increased the likelihood of TB – MM (p = 0.001). Subjects identified as whites were more prevalent among the TB – MM group (1.3%, p = 0.044) (Table 1).

**Table 1:** Distribution of socio-demographic and health history characteristics of tuberculosis cases according to multimorbidity status in Brazil, 2011

**Characteristics**		**TB (%)**	**TB – MM (%)**	p value*
Gender (39,878)	Female	12,645 (98.5)	190 (1.5)	<0.001
	Male	26,779 (99.0)	264 (1.0)	
Age (38,866)	<20 years	3,296 (99.9)	3 (0.1)	<0.001
	20 –39 years	17,780 (99.7)	54 (0.3)	
	40-59 years	12,898 (98.5)	192 (1.5)	
	> 60 years	5,438(96.4)	205 (3.6)	
Skin color (36,546)	White	13,953 (98.7)	182 (1.3)	0.044
	Non-White	22,174 (98.9)	2370 (1.1)	
School level (22,704)	Illiterate	1,936 (98.3)	33 (1.7)	0.001
	1 – 4 years	7,402 (98.5)	107 (1.5)	
	5 – 8 years	6,825 (99.0)	67 (1.0)	
	> 8 years	6,279 (99.1)	55 (0.9)	
Area of residence (28,247)	Urban	24,804 (98.8)	293 (1.2)	0.087
	Rural	3,124 (99.2)	26 (0.8)	
Institutionalization (38,073)	No	33.691 (98.7)	425 (1.3)	<0.001
	Yes	3,938 (99.5)	19 (0.5)	
Alcoholism (36,223)	No	29,553 (98.8)	349 (1.2)	0.331
	Yes	6,238 (98.7)	83 (1.3)	
HIV (26,335)	Negative	21,370 (98.8)	259 (1.2)	0.005
	Positive	4,672 (99.3)	34 (0.7)	

*MM* multimorbidity, *HIV* Human immunodeficiency virus, *TB* Tuberculosis.

*Pearson chi-square test.

Table 2 shows that there was no statistically significant difference between groups according to treatment type (p = 0.244) and TB form (p = 0.073).On the other hand, the proportion of those with positive tuberculin skin test was higher among those who did not have MM (99.3% TB vs 0.7% TB – MM p = 0.003).

**Table 2:** Distribution of clinical characteristics of tuberculosis cases according to multimorbidity status in Brazil, 2011

**Characteristics**		**TB**	**TB – MM**	p value*
		**n (%)**	**n (%)**	
Type of treatment (39,879)	New case	32,478 (98.9)	373 (1.1)	0.244
	Relapse	2,232 (98.5)	33 (1.5)	
	Return after abandonment	2,514 (99.2)	21 (0.8)	
	Unknown	84 (100)	0 (0)	
	Transferred	2,117 (98.7)	27 (1.3)	
TB form (39, 865)	Pulmonary	33,213 (98.9)	372 (1.1)	0.073
	Extrapulmonary	4,970 (98.6)	72 (1.4)	
	Pulmonary + extrapulmonary	1,228 (99.2)	10 (0.8)	
Tuberculin skin test (6,532)	Negative	1,868 (98.6)	27 (1.4)	0.003
	Positive	4,606 (99.3)	31 (0.7)	
X-ray suspicious (33,297)	No	2,220 (98.6)	32 (1.4)	0.218
	Yes	30,693 (98.9)	352 (1.1)	
Initialbacilloscopy (32,804)	Negative	8,721 (98.9)	101 (1,1)	0.964
	Positive	23,706 (98.8)	276 (1.2)	
Culture (9,667)	Negative	3,389 (99.1)	31 (0.9)	0.207
	Positive	6,173 (98.8)	74 (1.2)	
Histopathologic examination (4,298)	Not suggestive	260 (98.9)	3 (1.1)	0.084
	AFB positive	1,787 (98.9)	19 (1.1)	
	Suggestive of TB	2,187 (98.1)	42 (1.9)	
DOTS indication (37,432)	No	15,522 (98.8)	187 (1.2)	0.348
	Yes	21,487 (98.9)	236 (1.1)	
Under DOTS (33,504)	No	14,838 (98.8)	178 (1.2)	0.347
	Yes	18,289 (98.9)	199 (1.1)	
Occupational disease (24,077)	No	23,177 (98.9)	281 (1,1)	0.831
	Yes	611 (98.7)	8 (1.3)	
Treatment outcome (39,879)	Cured	28,625 (98.9)	302 (1.1)	<0.001
	Abandonment	5,490 (99.5)	29 (0.5)	
	Death from TB	2,243 (98.1)	44 (1.9)	
	Death from other cause	2,828 (97.4)	76 (2.6)	
	MDR TB	239 (98.8)	3 (1.2)	

*AFB* Acid fast bacilli, *DOTS* Directly observed treatment short, *MDR TB* Multidrug resistant tuberculosis, *MM* Multimorbidity, *TB* Tuberculosis.

*Pearson chi-square test.

The proportion of histopathological examination suggestive of TB was 98.1% for the subjects with TB and 1.9% for those with TB – MM (p = 0.084).

There were no differences with respect to DOTS program coverage (p = 0.348 DOTS indication and p = 0.347 DOTS realization) and occupational TB proportion (p = 0.831).

The hierarchical multivariable model (Table 3) shows that in the first level those subjects in the age group 40 – 59 years and those ≥ 60 years were more likely to develop TB – MM (OR = 17.89, 95% CI 5.71 – 56.03 and OR = 44.11, 95% CI 14.09 – 138.07, respectively). The odds of having TB – MM was lower among males (OR = 0.63, 95% CI 0.52 – 0.76).

**Table 3:** Hierarchical multivariable analysis of the association of multimorbidity status and characteristics of subjects with tuberculosis in Brazil, 2011

Covariates*		**OR (95% CI)**
Level 1		
Gender	Female	1.00
	Male	0.63 (0.52-0.76)
Age	<20 years	1.00
	20 –39 years	3.59 (1.12-11.48)
	40-59 years	17.89 (5.71-56.03)
	> 60 years	44.11 (14.09-138.07)
Skin color	White	1.00
	Non-white	0.88 (0.72-1.06)
Level 2		
School level	Illiterate	1.00
	1 – 4 years	1.32 (0.89-1.96)
	5 – 8 years	1.44 (0.93-2.21)
	> 8 years	1.31 (0.84-2.05)
Institutionalization	No	1.00
	Yes	0.59 (0.23-0.80)
Area of residence	Urban	1.00
	Rural	0.63 (0.42-0.95)
Level 3		
HIV	Negative	1.00
	Positive	0.75 (0.52-1.08)
Level 4		
TB form	Pulmonary	1.00
	Extrapulmonary	1.11 (0.83-1.47)
	Pulmonary + extrapulmonary	0.72 (0.38-1.36)
Histopathologic examination	Not suggestive of TB	1.00
	AFB positive	0.96 (0.28-3.28)
	Suggestive of TB	1.53 (0.46-5.03)
Level 5		
Treatment outcome	Cured	1.00
	Abandonment	0.73 (0.50-1.07)
	Death from TB	1.18 (0.86-1.64)
	Death from other cause	1.76 (1.36-2.28)
	MDR TB	1.33 (0.42-4.21)

*AFB* Acid fast bacilli, *MDR TB* Multidrug Resistant Tuberculosis, *MM* Multimorbidity, *HIV* Human immunodeficiency virus, *TB* Tuberculosis.

*Covariates with p < 0.10followed to next level of hierarchical model.

In the second level the odds of TB – MM was lower among subjects institutionalized (OR = 0.59, 95% CI 0.23 – 0.80). Death from causes other than TB was higher for subjects who had MM (OR = 1.76, 95% CI 1.36 – 2.28) (Table 3).

Concerning TB treatment outcome, 302 (66.5%) of 454 TB subjects with MM were classified as having been cured and 152 (33.5%) as not cured (abandonment, death from TB, death from other cause than TB and development of MDR TB).

The proportion of subjects who were considered as not cured was higher among males (females: 27.9%; males: 37.5%; p = 0.032), oldest age group (< 20 years: 33.3%; 20 – 39 years: 31.5%; 40 – 59 years: 26.6%; and ≥ 60 years: 40.5%; p = 0.032). Treatment outcome was not related to schooling (p = 0.217) and skin color (p = 0.065). Subjects who were not cured were more likely to be infected with HIV (41.1% among cured and 58.9% among not cured, p < 0.001) (Table 4).

**Table 4:** Distribution of socio-demographic and health history characteristics of cases of tuberculosis with multimorbidity according to TB treatment outcome status in Brazil, 2011

**Characteristics**		**Cured**	**Notcured**	**p value**
		**n (%)**	**n (%)**	
Gender (454)	Female	137 (72.1)	53 (27.9)	0.032*
	Male	165 (62.5)	99 (37.5)	
Age (454)	<20 years	2 (66.7)	1 (33.3)	0.032**
	20 –39 years	37 (68.5)	17 (31.5)	
	40-59 years	141 (73.4)	51 (26.6)	
	> 60 years	122 (59.5)	83 (40.5)	
Skin color (419)	White	111 (60.9)	71 (39.1)	0.065*
	Non-white	165 (69.6)	72 (30.4)	
School level (267)	Illiterate	19 (57.6)	14 (42.4)	0.217*
	1 – 4 years	71 (66.4)	36 (33.6)	
	5 – 8 years	48 (71.6)	19 (28.4)	
	> 8 years	42 (76.4)	13 (23.6)	
	Not applicable	2 (40.0)	3 (60.0)	
Area of residence (319)	Urban	195 (66.5)	98 (33.5)	0.280*
	Rural	20 (76.9)	6 (23.1)	
Institutionalization (444)	No	290 (68,2)	135 (31.8)	0.018*
	Yes	8 (42.1)	11 (57.9)	
HIV (293)	Negative	192 (74.1)	67 (25.9)	<0.001*
	Positive	14 (41.1)	20 (58.9)	
Alcoholism (432)	No	238 (68.2)	111 (31.8)	0.583*
	Yes	54 (65.1)	29 (34.9)	

*MM* Multimorbidity, *HIV* Human immunodeficiency virus, *TB* Tuberculosis.

*Pearson chi-square test.

**Likelihood-ratio chi-square test.

Table 5 describes the subjects with MM according to characteristics of TB presentation and TB treatment outcome status. Return for TB treatment after abandonment was higher among those who were not cured (28.6% cured vs 71.4% not cured, p < 0.001). The proportion of subjects who had a positive initial bacilloscopy test result, positive culture, x-ray suspicious of TB and TB presentation were similar between the two groups. Culture examination was not performed for most of the patients (77% of sample).

**Table 5:** Distribution of clinical characteristics of cases of tuberculosis with multimorbidity according to TB treatment outcome status in Brazil, 2011

**Characteristics**		**Cured**	**Not cured**	**p value**
		**n (%)**	**n (%)**	
Type of treatment (454)	New case	265 (71.1)	108 (28.9)	<0.001*
	Relapse	18 (54.5)	15 (45.5)	
	Return after abandonment	6 (28.6)	15 (71.4)	
	Transferred	13 (48.1)	14 (51.9)	
TB form (454)	Pulmonary	250(67.2)	122 (32.8)	0.508*
	Extrapulmonary	47 (65.3)	25 (34.7)	
	Pulmonary + extrapulmonary	5 (50.0)	5 (50.0)	
Tuberculin skin test (58)	Negative	15 (55.5)	12 (44.5)	<0.001**
	Positive	30 (96.8)	1 (3.2)	
X-ray suspicious of TB (384)	No	17 (53.1)	15 (46.9)	0.136*
	Yes	234 (66.5)	118 (33.5)	
Initial bacilloscopy (377)	Negative	63 (62.4)	38 (37.6)	0.295*
	Positive	188 (68..1)	88 (31.9)	
Culture (105)	Negative	17 (54.8)	14 (45.2)	0.271*
	Positive	49 (66.2)	25 (33.8)	
Histopathologic examination (64)	Not suggestive of TB	0 (0)	3 (100)	0.015**
	AFB positive	8 (42.1)	11 (57.9)	
	Suggestive of TB	28 (66.7)	14 (33.3)	
Under DOTS (377)	No	112 (62.9)	66 (37.1)	0.009*
	Yes	150 (75.4)	49 (24.6)	
Occupational disease (289)	No	193 (68.7)	88 (31.3)	0.698**
	Yes	6 (75.0)	2 (25.0)	

*AFB* Acid fast bacilli, *DOTS* Directly observed treatment short, *MM* Multimorbidity, *TB* Tuberculosis.

*Pearson chi-square test.

**Likelihood-ratio chi-square test.

The proportion of subjects under the DOTS program was lower among those considered as not cured (75.4% cured vs 24.6% not cured, p = 0.009). There were no statistically significant differences in occupational TB exposure (p = 0.698).

The hierarchical multivariate model is shown in Table 6. It shows that males were more likely to be considered as not cured at the end of TB treatment (OR = 1.55, 95% CI 1.04 – 2.32). In the second level, the odds of being not cured was higher (OR = 2.85, 95% CI 1.12 – 7.28) in those who were institutionalized. The odds of being not cured was higher among HIV positive subjects (OR = 3.93, 95% CI 1.86 – 8.30). Finally, in the fourth level of hierarchical logistic model, return to TB treatment after previous abandonment and transfer of treatment site were higher for subjects who were not cured compared to those who were (OR = 7.53, 95% CI 2.58 – 21.97 and OR = 2.76, 95% CI 1.20 – 6.38, respectively).

**Table 6:** Hierarchical multivariate analysis of the association of TB treatment outcome status and characteristics of subjects with tuberculosis and multimorbidity in Brazil, 2011

Characteristics*		**OR (95% CI)**
Level 1		
Gender	Female	1.00
	Male	1.55 (1.04-2.32)
Age	<20 years	1.00
	20 –39 years	1.06 (0.09-12;69)
	40-59 years	0.85 (0.07-9.69
	> 60 years	1.64 (0.14-18.62)
Skin color	White	1.00
	Non-white	0.71 (0.47-1.08)
Level 2		
Institutionalization	No	1.00
	Yes	2.85 (1.12-7.28)
Level 3		
HIV	Negative	1.00
	Positive	3.93 (1.86-8.30)
Level 4		
Type of TB treatment	New case	1.00
	Relapse	1.81 (0.82-4.01)
	Return after abandonment	7.53 (2.58-21.97)
	Transferred	2.76 (1.20-6.38)
DOTS realized	No	1.00
	Yes	0.48 (0.29-0.78)

*DOTS* Directly observed treatment short, *MM* Multimorbidity, *HIV* Human immunodeficiency virus, *TB* Tuberculosis.

*Covariates with p < 0.01 followed to next level of hierarchical model.

## Discussion

Prevalence of MM has wide variations across studies reported worldwide ranging from about 1% – 70% in general population. These variations are generally related to methods of MM classification [11]. The proportion of 1.4% (95% CI, 1.04 – 1.25%) of MM among subjects with TB found in this study is considered low, if compared with broad criteria of MM classification use in this study (≥ 2 morbidities). However, we emphasize here that there are no other estimates of MM proportion in a similar TB population.

In our study, subjects with MM tended to be older, female and have higher mortality. They were also less likely to live in rural areas, and have been institutionalized (prison, shelter, orphanage and others). Among TB – MM subjects, those who were not cured were more likely to be male, to be institutionalized, to have HIV infection; and to start TB treatment after previous abandonment and transfer of treatment site. They were also less likely to be under DOTS.

The sample size, the utilization of data based on an information system whose quality was confirmed in previous studies, and the absence of studies with a similar population are the main strengths of the present study.

However, some limitations should be mentioned. MM prevalence may have been underestimated because the information on MM was based on an optional entry in the database. We also performed a dichotomous classification of MM (no/yes) without considerations about severity of diseases. Furthermore, there were missing information that were not negligible. Nevertheless, our sample size still allowed us to maintain a high statistical power. Although we recognize that there is no statistical gain, we chose to work with all subjects without MM as comparison group since the database was structured and would not have involved additional direct assessments.

The conceptual model of simultaneous occurrence of multiple diseases in the same subject includes, among their causes, the age of populations, genetics and biological risk factors, life style, social environment, physical environment and health care support [17]. The consequences of MM include increase in mortality, impaired functional status, decreased of quality of life, complications of treatment and higher utilization of health care system [17]. In this study we compared one of those consequences – TB occurrence due to impaired functional status, with some causes and others consequences as described below.

The association between MM and age is strong and well recognized [11,18]. MM prevalence in elderly people may reach 71.8% [11]. Furthermore TB control in the elderly remains a challenge because of the limitations of the existing tools for the diagnosis and treatment of MM [7]. These data agree with our findings in which subjects ≥ 60 years had 44 times more chances of have TB and MM.

Gender type is associated with both TB [6] and MM [11]. It is postulated that men develop TB more often [6], but MM is more frequent in women [18]. We showed that simultaneous occurrence of TB and MM was lower for males [6,19].

Subjects identified as white were more prevalent among TB subjects with MM. This finding disagrees with a study performed in United States where nonwhites were more than seven times more likely than whites to acquire TB in general population; the age-adjusted risk of TB was especially high among Asians [20]. In Brazil, the diagnosis of disease other than TB may be more common in those with greater access to health care services.

The proportion of subjects with > 8 years of schooling was higher among those in the TB-only group, compared to the TB – MM group. Level of education is related to a socioeconomic condition of an individual, which can affect the probability of having TB [21] and MM [12,13].

No differences in treatment type and TB form or tuberculin skin test were found between the groups, although these factors are important in the disease diagnostic process [22].

The higher mortality from causes other than TB among subjects who had MM was expected. MM is associated with high mortality, reduced functional status, and increased use of both inpatient and ambulatory health care [23]. Subjects with MM may be prone to treatment failure and TB-drug intolerance and therefore, need a longer duration of treatment. However at the end of the ninth month of TB treatment (time of standard treatment) only one (0.2%) and twelve (2.6%) of the subjects with TB and TB-MM.

Concerning the treatment outcome, the proportion of subjects with MM who were considered as cured was below the recommended target of least 85% by the WHO [24].

The proportion of subjects who were considered as not cured was higher for males and older subjects. Male gender is associated with appearance of the disease and poor outcomes in TB patients [25]. As expected, older groups were found to have more advanced disease at the time of diagnosis, and a higher proportion had comorbidities. They also had significantly higher mortality compared with the younger age groups [26].

The institutionalized subject deserves attention in monitoring the evolution of TB, since this factor may act as an impediment in the treatment of the disease [27]. In this study, such issue was more evident in other institutions than prisons and psychiatric hospitals. It was most common in subjects institutionalized in rehabilitation clinics where the alcohol and other drugs abuse may affect treatment adherence and slows or prevent disease cure [28,29]. This reinforces the importance of partnership between the TB Control program and Mental Health program with the aim of incorporating intervention measures to align TB and addiction treatment [28].

Subjects with HIV infection were less likely to achieve TB cure, most likely due to impaired immunity [30] and the possible interaction between medications used in the treatment of AIDS and TB [31]. With respect to subjects who did not undergo HIV testing and were not cured, it is assumed that the fact of not having known their immunological status may hide the existence of immunosuppression. Similar findings have also been reported in other studies [29,32].

Those subjects who resumed treatment had a lower chance of achieving TB cure and subjects who undergo treatment during transfer also contribute to adverse treatment outcome in this study and agree with fidings among HIV subjects [33]. Nevertheless these associations were not found among Brazilian diabetics subjects [34].

## Conclusions

Tuberculosis remains a disease of disparity. Socio-economic determinants such as poverty, food insecurity, malnutrition and overcrowding besides being risk factors for TB infection, are also related to poor TB treatment outcome [3]. MM also increases this iniquity contributing to unfavourable clinical outcomes and treatment response in patients with TB.

In Brazil the recent-implemented policy to supplement income for vulnerable patients called “Brazil without poverty” is expect to reach 16 million people in the next five years [35]. With this program we expect to see an impact resulting in decline in TB and TB – MM prevalence among the poor and disadvantaged.

## Abbreviations

AIDS: Acquired immunodeficiency syndrome; AFB: Acid fast bacilli; CI: Confidence interval; DOTS: Directly observed treatment short; HIV: Human immunodeficiency virus; MDR TB: Multidrug resistant tuberculosis; MM: Multimorbidity; OR: Odds ratio; PPD: Purified protein derivative; SINAN: Brazilian National Surveillance System; TB: Tuberculosis; TB MM subjects: Tuberculosis and multimorbidities subjects; TB subjects: Tuberculosis without multimorbidities subjects.

## Competing interests

The authors declare that they have no competing interests.

## Authors’ contributions

BR conceived of the study, carried out the analyses and drafted the Results, Discussion, and Conclusions. TG drafted the Introduction, Methods, Discussion, and Conclusions. LM designed the tables and drafted the Discussion and Conclusions. BH made substantial contributions to the interpretation of data and revised the manuscript critically for important intellectual content. LR revised the manuscript critically for important intellectual content. EM made substantial contributions to the conception, design and interpretation of data and revised the manuscript critically for important intellectual content. All authors read and approved the final manuscripts.

## References

[B1] Uijen AA van de Lisdonk EH Multimorbidity in primary care: prevalence and trend over the last 20 years Eur J Gen Pract 2008 14 Suppl 1 28 32 18949641 10.1080/13814780802436093

[B2] Van den Akker MBF Roos S Knottnerus JA Comorbidity or multimorbidity: what’s in a name? A review of the literature Eur J Gen Pract 1996 2 65 70 10.3109/13814789609162146

[B3] Hargreaves JR Boccia D Evans CA Adato M Petticrew M Porter JD The social determinants of tuberculosis: from evidence to action Am J Public Health 2011 101 4 654 662 10.2105/AJPH.2010.199505 21330583 PMC3052350

[B4] Maciel EL A Promoção da Saúde e os Determinantes Socais da Tuberculose: Elementos para a ação. Promoção da Saúde na diversidade humana e na pluralidade de itinerários terapeuticos 2012 Campinas: Saberes 429 448

[B5] Brasil. Ministerio. da Saude Secretaria de Vigilância em Saúde Departamento de Vigilância Epidemiológica 2011 Programa Nacional de Controle da Tuberculose Available from: http://portal.saude.gov.br/portal/arquivos/pdf/manual_de_recomendacoes_tb.pdf

[B6] Lawn SD Zumla AI Tuberculosis Lancet 2011 378 9785 57 72 10.1016/S0140-6736(10)62173-3 21420161

[B7] Mori T Leung CC Tuberculosis in the global aging population Infect Dis Clin North Am 2010 24 3 751 768 10.1016/j.idc.2010.04.011 20674802

[B8] van den Akker M Buntinx F Metsemakers JF Roos S Knottnerus JA Multimorbidity in general practice: prevalence, incidence, and determinants of co-occurring chronic and recurrent diseases J Clin Epidemiol 1998 51 5 367 375 10.1016/S0895-4356(97)00306-5 9619963

[B9] Britt HC Harrison CM Miller GC Knox SA Prevalence and patterns of multimorbidity in Australia Med J Aust 2008 189 2 72 77 18637770 10.5694/j.1326-5377.2008.tb01919.x

[B10] Guralnik JM Assessing the impact of comorbidity in the older population Ann Epidemiol 1996 6 5 376 380 10.1016/S1047-2797(96)00060-9 8915467

[B11] Fortin M Stewart M Poitras ME Almirall J Maddocks H A systematic review of prevalence studies on multimorbidity: toward a more uniform methodology Ann Fam Med 2012 10 2 142 151 10.1370/afm.1337 22412006 PMC3315131

[B12] Anderson RT Sorlie P Backlund E Johnson N Kaplan GA Mortality effects of community socioeconomic status Epidemiology 1997 8 1 42 47 10.1097/00001648-199701000-00007 9116094

[B13] Salisbury C Johnson L Purdy S Valderas JM Montgomery AA Epidemiology and impact of multimorbidity in primary care: a retrospective cohort study Br J Gen Pract 2011 61 582 e12 e21 10.3399/bjgp11X548929 21401985 PMC3020068

[B14] Selig L Kritski AL Cascao AM Braga JU Trajman A de Carvalho RM Proposal for tuberculosis death surveillance in information systems Rev Saude Publica 2010 44 6 1072 1078 10.1590/S0034-89102010000600012 21107505

[B15] Oliveira GP Pinheiro RS Coeli CM Barreira D Codenotti SB Mortality information system for identifying underreported cases of tuberculosis in Brazil Revista brasileira de epidemiologia = Brazilian journal of epidemiology 2012 15 3 468 477 10.1590/S1415-790X2012000300003 23090296

[B16] Fuchs SC Victora CG Fachel J Modelo hierarquizado: uma proposta de modelagem aplicada à investigação de fatores de risco para diarréia grave Revista de Saúde Pública 1996 30 168 178 9077016 10.1590/s0034-89101996000200009

[B17] Gijsen R Hoeymans N Schellevis FG Ruwaard D Satariano WA van den Bos GA Causes and consequences of comorbidity: a review J Clin Epidemiol 2001 54 7 661 674 10.1016/S0895-4356(00)00363-2 11438406

[B18] Fortin M Hudon C Haggerty J Akker M Almirall J Prevalence estimates of multimorbidity: a comparative study of two sources BMC Health Serv Res 2010 10 111 10.1186/1472-6963-10-111 20459621 PMC2907759

[B19] Zhang X Andersen AB Lillebaek T Kamper-Jorgensen Z Thomsen VO Ladefoged K Effect of sex, age, and race on the clinical presentation of tuberculosis: a 15-year population-based study AmJTrop Med Hyg 2011 85 2 285 290 10.4269/ajtmh.2011.10-0630 PMC3144827 21813849

[B20] Buskin SE Gale JL Weiss NS Nolan CM Tuberculosis risk factors in adults in King County, Washington, 1988 through 1990 Am J Public Health 1994 84 11 1750 1756 10.2105/AJPH.84.11.1750 7977912 PMC1615189

[B21] Teixeira ECC Costa JS O impacto das condições de vida e da educação sobre a incidência de tuberculose no rasil Rev Econ 2011 37 2 106 123

[B22] Conde MB Melo FA Marques AM Cardoso NC Pinheiro VG Dalcin Pde T III Brazilian Thoracic Association Guidelines on tuberculosis J Bras Pneumol 2009 35 10 1018 1048 19918635 10.1590/s1806-37132009001000011

[B23] Barnett K Mercer SW Norbury M Watt G Wyke S Guthrie B Epidemiology of multimorbidity and implications for health care, research, and medical education: a cross-sectional study Lancet 2012 380 9836 37 43 10.1016/S0140-6736(12)60240-2 22579043

[B24] WHO Global Tuberculosis Programme. Global tuberculosis control: Surveillance, planning, and financing 2005 WHO report Geneva: World Health Organization 108 111

[B25] Fatiregun AA Ojo AS Bamgboye AE Treatment outcomes among pulmonary tuberculosis patients at treatment centers in Ibadan, Nigeria Annals of African medicine. [Comparative Study] 2009 8 2 100 104 10.4103/1596-3519.56237 19805940

[B26] Chan-Yeung M Noertjojo K Tan J Chan SL Tam CM Tuberculosis in the elderly in Hong Kong Int J Tuberc Lung Dis. [Comparative Study] 2002 6 9 771 779 12234132

[B27] Coninx R Maher D Reyes H Grzemska M Tuberculosis in prisons in countries with high prevalence BMJ 2000 320 7232 440 442 10.1136/bmj.320.7232.440 10669453 PMC1117551

[B28] Suhadev M Thomas BE Raja Sakthivel M Murugesan P Chandrasekaran V Charles N Alcohol use disorders (AUD) among tuberculosis patients: a study from Chennai, South India PLoS One 2011 6 5 e19485 10.1371/journal.pone.0019485 21611189 PMC3096635

[B29] Garrido Mda S Penna ML Perez-Porcuna TM de Souza AB Marreiro Lda S Albuquerque BC Factors associated with tuberculosis treatment default in an endemic area of the Brazilian Amazon: a case control-study PLoS One 2012 7 6 e39134 10.1371/journal.pone.0039134 22720052 PMC3373579

[B30] Swaminathan S Nagendran G HIV and tuberculosis in India J Biosci 2008 33 4 527 537 10.1007/s12038-008-0071-2 19208978

[B31] Dooley KE Kim PS Williams SD Hafner R TB and HIV therapeutics: pharmacology research priorities AIDS Res Treat 2012 2012 874083 22829999 10.1155/2012/874083 PMC3398575

[B32] Yone EW Kuaban C Kengne AP HIV testing, HIV status and outcomes of treatment for tuberculosis in a major diagnosis and treatment centre in Yaounde, Cameroon: a retrospective cohort study BMC Infect Dis 2012 12 190 10.1186/1471-2334-12-190 22894713 PMC3482584

[B33] Takarinda KC Harries AD Srinath S Mutasa-Apollo T Sandy C Mugurungi O Treatment outcomes of adult patients with recurrent tuberculosis in relation to HIV status in Zimbabwe: a retrospective record review BMC Publ Health 2012 12 124 10.1186/1471-2458-12-124 PMC3305664 22329930

[B34] Reis-Santos B Locatelli R Horta BL Faerstein E Sanchez MN Riley LW Socio-demographic and clinical differences in subjects with tuberculosis with and without diabetes mellitus in Brazil - a multivariate analysis PLoS One 2013 8 4 e62604 10.1371/journal.pone.0062604 23638123 PMC3634755

[B35] BRASIL GF Plano Brasil Sem Miséria 2011 Brasília: Governo Federal Available from: http://www.brasilsemmiseria.gov.br/

